# Socio-Psychological and External Factors Influencing Biosecurity Compliance in U.S. Poultry Farming

**DOI:** 10.3390/vetsci12100925

**Published:** 2025-09-24

**Authors:** Pedro Moura, Susanne Küker, Morgan Farnell, Julie Stowell-Moss, Jimmy Tickel, Patrik Buholzer, Heather L. Simmons

**Affiliations:** 1 SAFOSO AG, CH-3097 Liebefeld, Switzerland; 2 Department of Poultry Science, Texas A&M AgriLife Research, College Station, TX 77843, USA; 3 Institute for Infectious Animal Diseases, Texas A&M AgriLife Research, College Station, TX 77843, USA; 4 TAFS Forum, CH-3097 Liebefeld, Switzerland

**Keywords:** poultry, farmers, compliance, biosecurity, social, psychological-factors

## Abstract

Understanding why poultry farmers follow or neglect biosecurity practices is key to improving communication about the control of infectious and notifiable diseases like avian influenza. This study explored how socio-psychological, demographic, and contextual factors influence biosecurity compliance among U.S. poultry farmers. A questionnaire distributed at a poultry industry event yielded 67 responses from farmers, which were analyzed using a multivariable logistic regression with backward elimination. The predictors of high biosecurity tested included perceived outbreak impact, farming experience, and reliance on different information sources to form opinions on disease control. The final model showed that farmers who perceived that a disease outbreak would have a low impact were significantly less likely to follow strict biosecurity measures than those perceiving a higher impact (OR = 0.19, 95% CI [0.036, 0.925]). While compliance with biosecurity was high for most participants, certain practices, such as limiting flock outdoor access or contact with wild birds, were less commonly applied. Further research is needed to identify neglected biosecurity practices and the barriers to their implementation. Notable variations in farmers’ engagement with information sources suggest that communication strategies should involve trusted intermediaries to enhance risk awareness and biosecurity implementation support.

## Simple Summary

Protecting poultry farms from disease outbreaks is essential to safeguard animal health, public health, and farmers’ livelihoods. This study explored the factors influencing U.S. poultry farmers to follow disease prevention recommendations. We found that farmers who perceive a disease outbreak as a serious threat to their farm’s finances or reputation are more likely to adopt strong preventive measures, while those who do not see them as significant threats tend to be less strict. While overall compliance with disease prevention measures was high, some measures, such as preventing contact between farm birds and wild birds, were less commonly adopted. This may reflect operational challenges or limited understanding of their importance and implementation. Farmers form their opinions about disease risks in different ways, some rely on their experience or self-initiated research utilizing official and industry sources, while others seek guidance from advisors. To improve disease prevention, it is crucial for farmers to comprehend both the risks and potential impacts of diseases. Supplying tailored information that addresses their specific needs can enhance their understanding. By recognizing these factors, more effective disease communication programs can be developed, helping reduce the spread of poultry diseases and provide better support for farmers and the wider community.

## 1. Introduction

Since its introduction in North America, Highly Pathogenic Avian Influenza (HPAI) has led to the depopulation of more than 175 million poultry across the United States (U.S.), causing substantial economic losses [1,2,3,4,5]. To mitigate the impact of HPAI, intensified efforts are essential for preventing disease spread within the U.S.’s poultry sector and beyond [6,7], particularly in light of the ongoing HPAI disease pressure [5,7,8,9].

Farm level biosecurity measures play a critical role in reducing the risk of HPAI introduction and spread [10,11,12]. Strict adherence to biosecurity protocols, such as maintaining clear hygiene procedures upon entering poultry houses, is crucial to limiting the spread of the HPAI virus within poultry populations [11,13]. However, global research highlights that, due to multiple factors, adherence to essential biosecurity measures among poultry producers and farm staff varies, despite their importance for disease prevention [13,14,15,16,17].

Poultry farmers are a heterogeneous group with varying motivations for implementing biosecurity measures, that are shaped by both personal and external factors [18]. External factors define the broader environment where farmers operate and can significantly influence their behavior [19]. These include regulatory compliance, industry standards, and operational constraints such as time limitations and infrastructural deficiencies [11,18,20]. As demonstrated by a large-scale European study, biosecurity practices are closely associated with farm characteristics, including farm type, species reared, degree of specialization within the production chain, level of professionalism, and dependence on corporate contracts [11].

At an individual level, the farmers’ decisions to adopt biosecurity measures are influenced by various socio-psychological factors, internal psychological and social dynamics that shape their perceptions of disease prevention and control strategies [21]. These internal factors ultimately guide their decision-making processes [22,23]. Key socio-psychological factors include farmers’ knowledge of diseases and protective biosecurity measures [24,25,26] and their perception of the risk posed by a disease, or the impact this may have on their activity [27,28].

Research on farmers’ preventive actions against diseases reveals a consensus regarding the influence of specific socio-psychological factors [29], likely due to the consistent examination of similar behavioral constructs [30,31]. In addition, demographic characteristics, such as farming experience, have been shown to play a role in farmers’ behavior, with particularly strong effects observed among those who have previously faced the consequences of major disease outbreaks [23,27,32]. Providing information about disease prevention is fundamental to shape farmers’ perceptions and, ultimately, their actions. However, its effectiveness depends on how farmers with diverse backgrounds perceive the relevance and value of that information [20,25,32,33,34,35]. Therefore, it is imperative to consider whether the resources available to poultry producers in the U.S. are tailored to effectively engage diverse audiences in adopting biosecurity measures.

In the U.S., various resources are available to help poultry producers understand and implement biosecurity measures [36,37,38]. Of central importance, among them, is the National Poultry Improvement Plan (NPIP) Biosecurity Principles audit form, which establishes the national biosecurity standards and outlines essential management practices to prevent the introduction and spread of infectious diseases, including HPAI, across farms of all sizes and major poultry species [39]. Earlier in 2025, the USDA reinforced these efforts by unveiling a comprehensive $1 billion strategy to combat HPAI, with a focus on strengthening biosecurity, advancing research, and increasing support for farmers [40].

Given the diversity of farms within the U.S. poultry sector [41], variation in compliance with biosecurity standards is expected [42,43]. These differences arise not only from farm-specific factors, such as the type of animals kept and the level of professionalism, but also from internal-level differences in perceptions and behaviors patterns among poultry farmers [19]. To develop more effective strategies for promoting biosecurity, it is therefore central to identify those farmer groups of who generally operate to lower biosecurity standards due to external constraints, such as limited resources or infrastructure challenges [11,20]. At the same time, it is also important to understand the farmers’ internal decision-making processes and motivations in relation to biosecurity implementation [29]. As opposed to broad guidelines, tailored advice that accounts for both the farm context and individual motivational factors is more likely to be perceived as relevant and, ultimately, adopted [20,25,34].

While several studies (conducted in European countries, Canada, and parts of Asia) have explored the influence of socio-psychological factors on biosecurity adoption [24,27,28,32,33,35,44], as well as the role of farm characteristics and external influences [11,18,19,20], evidence from the U.S. remains limited.

This study examines how selected external factors, such as farm characteristics, demographic characteristics, and socio-psychological factors influence biosecurity-related decision-making among U.S. poultry farmers. Conducted as part of a broader initiative funded by the United States Department of Agriculture (USDA), this research aims to support the development of tailored communication strategies and educational resources that enhance compliance and reduce the risk of future disease outbreaks.

## 2. Materials and Methods

### 2.1. Questionnaire Design and Structure

#### 2.1.1. Objectives and Scope

A questionnaire was developed to assess the influence of a selection of socio-psychological factors, demographic characteristics of farmers, and external factors such as farm/business characteristics on the adoption of biosecurity measures aimed at preventing HPAI. The primary objective of this questionnaire was to provide a concise and practical evaluation of the above-mentioned dimensions, rather than an exhaustive analysis of all possible contributing factors.

#### 2.1.2. Development Process

The questionnaire was designed in English to target poultry producers across the U.S. It was developed using the Qualtrics platform [45] and was tested nine times to ensure clarity and usability. It was structured into four sections, covering: demographic information, farm characteristics, socio-psychological factors and the implementation of biosecurity practices. The complete questionnaire can be found in Tables S1 and S2 of the Supplementary Materials.

#### 2.1.3. Demographics

This section included optional questions on gender and age, as well as a question about whether respondents operate their business within the U.S. or internationally. The latter question was included primarily to identify and exclude participants who operate exclusively outside of the U.S. and therefore were not relevant to the objectives of the study. This step was necessary, because the questionnaire was administered at the International Production and Processing Expo (IPPE) 2025, an international poultry industry event in the U.S. [46].

#### 2.1.4. Farm Characteristics

The questionnaire included four questions about farm characteristics: the type of poultry farmed (chickens, ducks, turkey, geese, quail, others), category of the farm (large, small or fancy bird producer), subcategory within large producers (egg production, broiler production, primary breeder, pullet farm and hatchery) and production model (independent producers or associated with a large company).

#### 2.1.5. Socio-Psychological Factors

The socio-psychological factors included in the questionnaire were identified through a scoping literature review focused on behavioral determinants of biosecurity adoption in livestock farming. Based on the frequency and consistency with which certain socio-psychological factors were cited, four key factors: farming experience, risk and impact perception, contextual influences, and knowledge were prioritized for inclusion due to their conceptual relevance, empirical support, and potential to inform policy or educational interventions. These factors capture both cognitive elements of the individual (e.g., risk perception) and external contextual factors (e.g., perceived responsibility towards public health). The consistent valorization of these factors in the literature on the topic indicates a consensus regarding their relevance and influence. Their inclusion also accounted for the practical feasibility of measurement with a questionnaire. Table 1 provides an overview of the socio-psychological factors evaluated, detailing the number of questions used to assess each of them, their significance, and the measurement methods applied. The table also cites relevant literature supporting their influence in disease prevention and control.

#### 2.1.6. Biosecurity

The biosecurity section of the questionnaire was primarily based on the NPIP Biosecurity Principles audit form. This form was developed after the 2014–2015 HPAI outbreaks in the U.S. and covers fundamental farm-level biosecurity measures to prevent the introduction and spread of infectious diseases [59]. Twenty-one questions representing the most critical biosecurity topics, selected based on the expert opinion of the authors, were prioritized for the final version of the questionnaire. Table 2 presents the number of questions by biosecurity topic.

### 2.2. Questionnaire Application

The questionnaire was made accessible via mobile device using a QR-code available during the IPPE 2025, in Atlanta, GA, USA. A dedicated booth was set up at the IPPE, including posters with a detailed project description. The booth remained active throughout all three days of the IPPE, with the consistent presence of three to four project members, available to clarify any questions. Passing exposition attendees were actively approached and invited to take part in the questionnaire. Upon completion, respondents had the opportunity to enter a prize drawing for small promotional gifts.

A total of 141 responses were collected during the IPPE. Entries were screened and excluded if over 50% of the items were unanswered, the respondent did not operate in the U.S., or the poultry species was unspecified. This ensured that only U.S.-based poultry farmers were included in the analysis.

#### 2.2.1. Biosecurity Scores

Each of the 21 biosecurity-related questions listed in Table S2 was operationalized into a specific variable representing a measurable farm practice. The variable names were derived directly from the keywords in each question. Each variable was assigned a score value from 1 to 4, with 4 representing the highest level of biosecurity compliance. Scoring methods varied by question format (e.g., Likert-type and binary) and were based on the alignment with the positive biosecurity practices. Full details are provided in Table 3.

For each respondent, an average overall biosecurity score was then calculated, excluding any skipped questions. These scores were then converted into a binary outcome variable, where values above 3.0 indicated high biosecurity implementation (binary = 1), and values of 3.0 or below indicated lower implementation (binary = 0).

#### 2.2.2. Predictor Variables

The questionnaire assessed key conceptual domains related to demographics, farm characteristics, and socio-psychological factors, using multiple questions within each domain. The questions presented in Table S1 (Supplementary Materials) were then used as independent predictors.

To minimize the exclusion of observations with missing predictor data and improve the statistical power of the full model, closely related numeric predictor variables (all on a scale from 0 to 10) were combined into composite predictors. Each element of the composite predictor was categorized depending on its score as Low (0–3), Medium (4–6), or High (7–10). To assign a categorical classification to these merged variables, the following hierarchical logic was followed:

- two high values yielded a high score;
- two medium values yielded medium score;
- two low values yielded low score;
- In mixed cases, high overruled medium or low, and medium overruled low;
- If only one value was available, its classification was retained.

Following this rationale the composite variables presented in Table 4 were created.

This approach ensured that strong perceptions in one area were preserved in the composite predictor.

### 2.3. Statistical Analysis

A stepwise logistic regression with backward elimination was conducted to examine the influence of demographic variables, farm characteristics, and socio-psychological factors on biosecurity compliance. All statistical analyses were performed using R (version 4.3.2) [60], with packages including tidyverse, lubridate, data.table, DescTools, janitor, officer, flextable, summarytools, readxl, corrplot, and corrr.

Before modeling, all predictors were processed according to their data type: categorical ones were converted to factors, and numerical were kept as continuous. To identify candidate predictors for the multivariable model, each of these was first evaluated using univariable logistic regression. Predictors with a significance level of *p* < 0.20 were selected for inclusion in the next step. Additionally, the predictor *age* was excluded at this stage due to a low response rate (*n* = 38), to preserve model stability.

To assess the potential for multicollinearity among categorical predictors, pairwise Chi-square tests were performed, and Cramér’s V coefficients were calculated. A Cramér’s V value greater than 0.50 (V > 0.50) was considered indicative of a strong association and potential redundancy.

The multivariable logistic regression analysis was then performed using a stepwise backward elimination approach. At each step, the predictor contributing the least to model fit, based on the Akaike Information Criterion (AIC), was removed. The final model included only predictors that improved model fit and achieved statistical significance (*p* < 0.05).

Throughout the model selection process, predictors removed from the model were evaluated for potential confounding effects. A change greater than 10% in the estimated coefficients of the remaining predictors following their removal was considered evidence of confounding.

Effect sizes were estimated from the logistic regression models and reported as odds ratios (ORs) with 95% confidence intervals (CIs), providing a quantitative measure of the strength and direction of associations between each predictor and the likelihood of high biosecurity compliance.

## 3. Results

After applying the exclusion criteria, 74 responses were excluded, resulting in a final sample of 67 U.S.-based poultry producers. A detailed descriptive summary of the analyzed dataset is provided in Table S3 (Supplementary Materials).

### 3.1. Survey Results: Demographics, Farm Characteristics, Socio-Psychological Factors and Biosecurity Topics

#### 3.1.1. Demographics

Among the respondents (*n =* 38/67), the mean age was 36.9 years, with a median age of 35.5 years. Gender distribution was balanced among respondents (*n =* 42/67), with 47.6% identifying as female and 50.0% as male; one respondent selected “prefer not to say”. A total of 81.5% (*n =* 53/65) of respondents classified themselves as domestic entities, while 13.8% (*n =* 9/65) as international entities operating in the U.S. and 4.6% (*n =* 3/65) reported not representing a business. The latter group clarified their status as backyard producers, hobby farmers, or small flock owners.

#### 3.1.2. Farm Characteristics

When describing their farm characteristics, 88.1% (*n =* 59/67) of respondents reported keeping chickens, while the remaining 11.9% (*n =* 8/67) kept turkeys, ducks, or multispecies flocks. The majority (61.2%, *n =* 38/62) categorized their operations as large-scale (more than 5000 birds), while 25.8% (*n =* 16/62) identified as small producers (fewer than 5000 birds), and 12.9% (*n =* 8/62) as ‘fancy bird’ producers. Among large producers, broiler (50.0%, *n =* 38/62) and egg (34.2%, *n =* 13/38) production were most common business operations. Regarding work independence, 66.7% (*n =* 38/57) of respondents reported operating independently, while 33.3% (*n =* 19/57) were associated with larger companies.

#### 3.1.3. Socio-Psychological Factors

Respondents reported varying levels of experience in poultry farming: 37.9% (*n =* 25/66) were five years or less, 19.7% (*n =* 13/66) had six to ten years, 13.6% had eleven to twenty years, and 28.8% (*n =* 19/66) reported more than twenty years of experience.

Regarding their perception of risk and impact, over one third of respondents (36.5%, *n =* 23/63) reported having faced major challenges related to poultry diseases. Among those, 21 provided additional details, HPAI was the most frequently mentioned disease, cited by 6 respondents. The most reported challenges were flock culling due to outbreaks, and issues with biosecurity implementation. The mean perceived economic impact on respondents’ businesses of a hypothetical outbreak of a disease such as HPAI was moderately high, with a mean score of 6.6 (SD = 3.1) on a 10-point scale (*n =* 63/67), while the mean perceived reputational impact was lower but still notable at 4.9 (SD = 3.0) (*n =* 58/67).

Concerning their perception of responsibility, participants generally expressed a strong, shared sense of responsibility among farmers for disease prevention, regarding the protection of public and animal health. On a scale from 0 to 10, the perceived responsibility toward the public health averaged 7.8 (SD = 2.4) (*n =* 65/67), and the responsibility toward the poultry sector averaged 7.3 (SD = 2.6) (*n =* 58/67).

Relating to their contextual influences, a total of 49.1% (*n =* 27/55) reported that their business partners required biosecurity standards exceeding those recommended by the national authorities. Of these, 84.6% (*n =* 22/26) indicated that they received assistance in implementing these higher standards.

Regarding knowledge and sources of information, participants were asked whether they received all the necessary information and advice regarding the measures required to prevent the introduction of diseases such as HPAI. The public sector, particularly national animal health authorities, was perceived as the most important source of information, with a mean rating of 7.5 (SD = 2.5, *n =* 65/67), followed by county extension agents (mean = 7.0, SD = 2.6, *n =* 65/67). The private sector, including cooperatives, federations, and producer associations, received a similar rating (mean = 7.1, SD = 3.1, *n =* 64/67), as did integrators (mean = 7.0, SD = 3.1, *n =* 63/67). Concerning the most influential opinions guiding decisions on disease prevention and control, the farmers’ own opinions, shaped by personal experience, emerged as the predominant influence cited by 47.0% (*n =* 31/66). An additional 12.1% (*n =* 8/66) reported forming their own opinions based on independent research, including consultation of scientific publications and agricultural magazines. Furthermore, 25.8% (*n =* 17/66) primarily valued advice from others such as veterinary authorities, private veterinarians, NPIP auditors, county extension personnel, or peer networks, while 15.2% (*n =* 10/66) relied on other external inputs, predominantly from industry and government agencies.

#### 3.1.4. Biosecurity Topics

Biosecurity measures were broadly adopted across the sample, reflecting a consistently high level of compliance. Over half of the respondents (56.9%, *n =* 33/58) indicated their businesses had previously undergone a NPIP audit. The majority (89.0%, *n =* 57/64) stated that they follow a biosecurity plan specifically designed for their operations, and 88.3% (*n =* 53/60) stated that this plan is reviewed every year.

Training practices related to biosecurity were also widely implemented, most respondents 85.4% (*n =* 41/48) stated that their staff had easy access to biosecurity training materials. Among those who provided additional information (*n =* 18), commonly mentioned formats included in-person sessions and customized instruction videos on the use of personal protective equipment and cleaning procedures.

In terms of farm infrastructure and contact with wild birds or other animals, outdoor access of poultry flocks was restricted in 54.5% (*n =* 36/66) of the operations. Control measures to prevent poultry from encountering wild birds, feces, or feathers were reported as consistently applied by 68.3% of respondents (*n =* 41/60). Additionally, 75.8% (*n =* 47/62) indicated that measures are always implemented to prevent exposure to rodents, insects and other animals.

Adherence to hygiene practices, including clothing protocol, disinfection procedures and measures for managing staff contact with other birds, was generally high. A total of 79.7% (*n =* 47/59) of respondents reported that staff always follow clear hygienic procedures when entering the poultry house(s). Additionally, 77.6% (*n =* 45/58) indicated that staff who had contact with other poultry species consistently follow specific procedures before re-entering the farm. Furthermore, 75.0% (*n =* 42/56) reported that non-farm personnel always adhere to a defined set of protective measures when entering the farm premises.

Concerning equipment disinfection and sharing practices, 76.2% (*n =* 48/63) of respondents reported that appropriate protocols are in place and are always applied. The use of shared equipment from other farms was prohibited in 82.8% (*n =* 53/64) of operations, while 57.6% (*n =* 34/59) did not allow equipment sharing between poultry houses within the same farm.

Regarding sourcing replacement poultry, 62.0% (*n =* 31/50) of operations that procure replacements reported that they consistently source birds from suppliers compliant with NPIP provisions.

Concerning flock contact with surface water and water supply, 66.1% (*n =* 41/62) reported that their flocks do not have direct access to surface water. Regarding the use of surface water, from the farmers who used this type of water source, 71.4% (*n =* 25/35) indicated that the water was treated prior to use.

Feed and bedding storage practices were also addressed. A total of 80.6% (*n =* 50/62) reported that feed ingredients are always stored in closed containers. Concerning bedding materials, 71.9% (*n =* 41/57) of respondents reported that they store these materials. Of those, 61.0% (*n =* 25/41) stated that bedding materials are always stored in closed containers.

Finally, concerning management of sick birds, 69.6% (*n =* 39/56) of respondents reported that all staff members were familiar with the appropriate protocols to follow when discovering a sick bird.

#### 3.1.5. Logistic Regression Analysis

Of the 67 respondents who met the inclusion criteria, 13 additional responses were excluded due to missing data on one or more predictor variables. The logistic regression analysis thus included 54 complete cases.

Based on the univariable logistic regression analysis results, four predictors: *independence*, *experience*, *impact_comp* and *opinion* met the inclusion threshold (*p* < 0.20) and were retained for further evaluation in the multivariable model. The results of the univariable regression analysis are presented in Table S4 (Supplementary Materials).

To assess potential redundancy among the selected predictors, a pairwise correlation analysis using Cramér’s V was performed. Associations were generally weak (V = 0.10–0.30). The strongest was observed between *experience* and *impact_comp* (V = 0.32), indicating a moderate association. Since no strong associations (V > 0.50) were found, all four predictors were retained for the multivariable analysis.

A multivariable logistic regression was then conducted to identify the most influential predictors of high biosecurity implementation. The initial model (Table 5) included the four previously selected predictors: *independence*, *experience*, *impact_comp*, and *opinion.* While none of these predictors reached statistical significance individually (*p* < 0.05), the full model fit demonstrated an improved fit over the null model (AIC = 63.5; residual deviance = 45.5 on 45 degrees of freedom), suggesting potential explanatory value.

To refine the model, a stepwise logistic regression with backward elimination was applied, removing predictors that contributed the least to model fit, based on AIC values. The final model retained a single predictor, *impact_comp* (combined perception of economic and reputational impact related to a disease outbreak) as the strongest determinant of high biosecurity implementation. During model refinement, a potential confounding effect was observed, removing the predictors’ *experience* (experience in farming) and *opinion* (how farmers form their opinions) altered coefficients of the remaining predictors, indicating they influenced both the other predictors and the outcome. However, these predictors were not reintroduced due to their conceptual overlap with perceived impact (*impact_comp*) and because model fit improved without them (lower AIC). Therefore, their influence is interpreted as indirectly captured through their association with perceived impact.

The final model (Table 6) showed that respondents who perceived the potential consequences of a disease outbreak as low were significantly less likely to implement high biosecurity standards compared to those perceiving a high impact (OR = 0.19, 95% CI [0.036, 0.925], *p* = 0.0417). Respondents with a medium impact perception also showed higher odds of implementing high biosecurity, although this association was not statistically significant (*p* = 0.3152). Overall, these findings suggest that the perceiving disease outbreaks as a minor economic or reputational threat is associated with the lower biosecurity implementation. Other factors such as experience, independence, and opinion showed no consistent or significant associations in the final model.

## 4. Discussion

The present study contributes to the growing body of evidence on how socio-psychological, demographic, and contextual factors influence the implementation of biosecurity in poultry production. One of the most compelling findings was the central role of perceived impact, farmers’ expectations of reputational and economic losses due to a disease outbreak, in predicting compliance with high biosecurity standards. Poultry farmers who perceived the potential impact of disease outbreaks as low were significantly less likely to consistently implement high biosecurity measures. This aligns with previous research showing that threat appraisal, particularly the anticipated economic and emotional consequences of disease, shapes farmers’ motivation to adopt preventive measures [15,22,23,61,62]. While the financial consequences of an outbreak, such as culling of animals and loss of market access, are often severe [61,62], farmers are often also driven by professional pride and personal principles, valuing good animal husbandry and the reputation of their operations [20,22,23,61]. Farmers’ perception of risk is often shaped by local disease incidence [22,24,49], with greater awareness of the risks and possible impacts when outbreaks occur in nearby operations [22,24,25,50]. In contrast, limited exposure or knowledge on the topic can lead to a risk underestimation [22,50], even in high-risk contexts, such as currently observed with HPAI in the U.S. [1,2,3,4]. Similar mechanisms have been reported among different types of poultry farmers in Bangladesh [33] and in Taiwan [19], suggesting that this cognitive pattern is relevant across regions and farm types. These observations align with the Protection Motivation Theory, which suggests that both threat and coping appraisals are of central importance for behavioral change [63]. Comparable findings in other sectors, such as European cattle production [32,34], further support the idea that low perceived vulnerability is a cross-sector barrier to biosecurity implementation.

In the present study, farming experience and operational independence showed limited predictive power for biosecurity compliance. While prior research in cattle farming has linked experience to more accurate risk and impact perception and stronger adherence to control measures [32,34], our data did not reveal statistically significant associations. One possible explanation is the high baseline of biosecurity in our sample, nearly 90% of respondents had a formal biosecurity plan and staff training in place. This level of professionalism may have masked the effects of background variables and reflect a sampling bias towards larger or more engaged producers, as reported in a similar study [44]. Nevertheless, as noted in the results section, a confounding relationship was observed between the predictors that captured experience in farming, how farmers form their opinion and overall perception of the impact. These predictors were excluded from the final model due to their conceptual overlap with the farmers’ perception of impact and their negative effect on model fit. Nevertheless, we interpreted the results with this overlap in mind, recognizing that the perception of a disease’s impact is a complex mental construct shaped by multiple socio-psychological factors, with farmers forming their risk and impact perceptions through an interplay of personal experience, external advice, and research [24,27,32,47].

Moreover, our data reveal considerable heterogeneity in farmers’ decision-making processes, with some forming their opinions on disease prevention by mainly relying on personal experience (47%), others on consultation with advisors (25.8%), and a minority on their own research, accessing scientific or government sources (15.2%). This diversity reflects broader challenges in risk communication: information about disease prevention is valued differently depending on its source, and is subsequently interpreted, and internalized as knowledge in diverse ways across farming audiences. Literature shows that that is how information is communicated, by whom and in what format, strongly influences farmer perceptions and ultimately, their actions [20,25,32,33,34,35]. In the case of HPAI prevention, the availability of information from authorities or industry actors is fundamental. However, meaningful change depends on how this information shapes the mental constructs related to the farmers’ decision-making logic [33,64,65]. These dynamics are consistent with findings from other livestock sectors where trust in the messenger and alignment with personal logic determine whether advice is adopted [32,50]. Veterinarians and other consultants are often regarded as trusted advisors, playing an important role in knowledge dissemination [50,58,61,66,67], in addition to media and government sources [24,50,64] which often provide the basis for discussions with advisors [24]. Tailoring messages related to HPAI prevention to different groups, such as backyard vs. commercial producers, is therefore essential, as access to information and motivational drivers differs [33]. As supported by our findings, a one-size-fits-all communication strategy may not be optimal for reaching the diverse population of farmers [20,25,34].

We also observed discrepancies between the implementation of basic hygiene practices (e.g., disinfection protocols) and more advanced measures such as restricting poultry access to the outdoors, minimizing contact with wild birds, their feces or feathers and preventing direct access to surface water, which were not so widely practiced [11,15]. The inconsistent adoption of measures known to limit the spread of HPAI raises important questions about the decision-making process behind these gaps. Farmers often weigh the anticipated benefits of a proposed biosecurity measure against its potential drawbacks [22]. The Health Belief Model, which highlights the importance of perceived benefits and barriers in behavior change, provides a useful lens for interpreting these findings [33,44,61,68,69]. Commonly cited barriers include cost, time requirements, and the perceived impracticality of certain measures [11,24,33,50]. On the other hand, farmers are more likely to adopt measures seen as effective and worthwhile [22,24,25,33,34,61]. As a result, practices seen as logistically challenging, expensive, or marginally beneficial are often overlooked, even when overall compliance appears high. In the context of HPAI, where disease pressure is often high, and the transmission pathways can be difficult to grasp, some farmers have reported feeling powerless in preventing outbreaks [19]. Compounding this, a Europe-wide study of large poultry farmers found that, amid the current high incidence of HPAI outbreaks, limited awareness or understanding of the expected benefits of biosecurity was a key reason for not implementing certain measures [11]. Other studies also note limited application of measures to prevent flocks from contact with wild birds, particularly in free-range farms or in operations with limiting structural characteristics, who often view such measures as not adapted to their operations [11,14]. Despite these challenges, these practices remain critical to limiting disease spread. While the USDA has developed risk-based reduction biosecurity materials [70], ensuring their effective dissemination and alignment with farmers’ decision-making processes is essential for improving implementation.

### 4.1. Study Limitations

The findings of this study should be interpreted considering the following limitations. First, the reliance on self-reported data about biosecurity practices introduces the potential for social desirability bias, meaning that farmers may report higher levels of compliance than they practice [32]. Second, participants were recruited from the IPPE, an industry event that likely attracted larger-scale poultry producers, that can be more exposed to biosecurity topics. This recruitment approach introduces a selection bias, suggesting that the study may not fully reflect the biosecurity practices of smaller or less proactive farmers. Additionally, the relatively small sample size (67 responses) limits the generalizability of the results. Future studies should aim to gather a larger and more diverse representative sample of farmers to better capture the range of biosecurity practices across different farm types. Additionally, a more independent assessment of biosecurity practices, preferably including farm visits, could provide a more robust evaluation of their implementation. Such farm visits were initially part of the original study design but could not be conducted due to strict biosecurity measures implemented during the HPAI outbreaks.

### 4.2. Implications for Future Interventions

Keeping in mind the limitations of the present study, future communication strategies to improve biosecurity compliance among U.S. poultry farmers must account for the complexity of their decision-making process. This requires consideration about farmers’ perceptions, motivations, and practical challenges in implementing biosecurity measures [19,22,25,32,34,54,68]. Tailored outreach then should focus on the key perceptions expected to influence farmers’ decisions and address the specific barriers and benefits related to critical biosecurity practices [20,22,32,34,54]. Addressing low risk perception through scenario-based risk visualization, can be particularly effective [24,61]. Leveraging a wide range of information sources is important to accommodate for different information-seeking behaviors [22]. However, particular emphasis should be placed on strengthening the role of key farm advisors [24,25,50,54]. These considerations are especially important when tailoring outreach to subgroups of farmers with structural disadvantages or entrenched behavioral patterns [71].

## 5. Conclusions

This study provides insights into the factors influencing biosecurity compliance among U.S. poultry farmers. To enhance biosecurity adoption, future communication efforts should be more nuanced and tailored to address the most impactful psychological factors, especially risk and impact perception, while also accounting for broader operational context influences. Additionally, variations in farmers’ engagement with different information sources and stakeholders must be considered. Communication efforts should mobilize trusted intermediaries, who can reinforce risk awareness and support the practical implementation of biosecurity measures. However, further research involving a more representative sample of farmers is necessary to identify critical biosecurity practices that remain under-implemented at the farm level and to explore the barriers hindering their adoption.

## Figures and Tables

**Table 1:** Number of questions related to socio-psychological factors, their significance, measurement methods, and relevant references.

Socio-Psychological Factors	Significance	Number of Questions	Measurement	References
Farming Experience	Indicator of accumulated practical knowledge	1	Years of experience	[19,24,27,32]
Risk and Impact perception	Awareness of and concern about the impact of avian diseases	3	Faced major avian disease challenges (yes/no) and impact description (open); Expected consequences of a disease outbreak on economic losses and reputation, Likert scales (1–10), with 10 being the highest perceived impact	[24,27,28,47,48,49,50]
Contextual Influences	Social motivations and operational environment	3	Responsibility for protecting public health and national poultry, Likert scales (1–10), with 10 representing the strongest sense of responsibility Business partners’ requirement for higher biosecurity standards (yes/no); Receipt of support for standard implementation (yes/no).	[20,24,32,35,51,52,53]
Knowledge	How commonly available information sources are valued and contribute to decision-making	2	Perceived value of biosecurity information from public and private sources, Likert scales (1–10), with 10 being the highest perception of value Reliance on different information sources to form opinions on disease control (options: own experience, own research, external inputs, consultation).	[19,24,25,26,27,32,47,54,55,56,57,58]

**Table 2:** Distribution of questions related to biosecurity topics.

Biosecurity Topic	Number of Questions
Biosecurity audit	1
Biosecurity planning	3
Training	2
Farm infrastructure	1
Clothes and disinfection	2
Personnel contacts with other birds	1
Contact with wild birds	1
Contact with rodents, insects, and other animals	1
Equipment	2
Replacement poultry—trusted sources	1
Water supply	2
Feed	1
Bedding materials	2
Sick birds report	1

**Table 3:** Biosecurity compliance scoring scheme by question format and variable.

Question Type	Scoring Scale	Variable Code
Likert-type evaluating the frequency/consistency of biosecurity practices	1 = No, and there is no intention/plans to do so 2 = No, but it is being considered 3 = Yes, but only sometimes 4 = Yes, always	staff_enter_hygienic; non_farm_personnel_hygienic; staff_poultry_contact; wild_birds; rodents_insects_others; farm_equipment_disinfection; replacement_poultry; feed_containers; bedding_materials_containers;
Likert-type evaluating the capacity of staff	1 = No, none of them do 2 = No, only some of them do 3 = Yes, most of them do 4 = Yes, all of them do	sick_bird_report
Binary (Yes/No) Positive scenario: Yes	Yes: 4 points No: 1 point	npip, biosecurity_plan, biosecurity_plan_review, staff_training, surface_water_treatment, bedding_materials_store
Binary (Yes/No) Positive scenario: No	Yes: 1 point No: 4 points	outdoor_access, shared_equipment_between_houses, shared_equipment_other_farms, surface_water

**Table 4:** Construction of composite predictor variables from thematically related items.

Grouped Predictors	Significance	Questions Merged
impact_comp	Perceived economic and reputational impacts of disease outbreaks.	How would you assess the impact on your business in the case of an outbreak of an avian disease, such as Avian Influenza? In terms of: Economic Losses + Reputation
responsibility_comp	Sense of shared responsibility for safeguarding public health and the poultry sector.	Do you agree that farmers are jointly responsible for preventing animal diseases to protect both: Public Health + Domestic avian population in your business
necessary_info_public_comp	Perceived adequacy and relevance of public sector biosecurity guidance	How much do you agree with the statement “I receive all the necessary information and advice about the measures that I need to take to prevent the introduction of avian diseases, such as Avian Influenza, into my farm, from the public sector”: National Animal Health Authorities + County Extension Agents
necessary_info_private_comp	Perceived adequacy and relevance of private sector biosecurity guidance	How much do you agree with the statement “I receive all the necessary information and advice about the measures that I need to take to prevent the introduction of avian diseases, such as Avian Influenza, onto my farm, from the private sector”: Cooperatives/Federations/Associations + Integrators

**Table 5:** Multivariable logistic regression, Initial model.

Predictor	Coefficient	Odds Ratio	95% Confidence Interval	Standard Error	*p* Value
(Intercept)	2.485	12	[1.13, 240.0]	1.34	0.065
independence_Independently	−0.499	0.607	[0.093, 3.40]	0.893	0.576
experience_20+ years	0.278	1.32	[0.1, 16.6]	1.25	0.823
experience_5–10 years	0.476	1.61	[0.087, 47.9]	1.49	0.75
experience_Up to 5 years	−0.685	0.504	[0.047, 4.34]	1.12	0.541
impact_comp_Low	−0.865	0.421	[0.067, 2.56]	0.912	0.343
impact_comp_Medium	1.411	4.1	[0.511, 89.7]	1.21	0.244
opinion_My own, based on experience	−1.211	0.298	[0.038, 1.63]	0.92	0.187
opinion_My own, based on research	−0.799	0.45	[0.024, 15.5]	1.54	0.604

**Table 6:** Multivariable logistic regression, refined model.

Predictor	Coefficient	Odds Ratio	95% Confidence Interval	Standard Error	*p* Value
(Intercept)	1.428	4.17	[1.83, 11.2]	0.455	0.002
impact_comp_Low	−1.650	0.192	[0.036, 0.925]	0.81	0.042
impact_comp_Medium	1.138	3.12	[0.463, 62.2]	1.13	0.315
(Intercept)	1.428	4.17	[1.83, 11.2]	0.455	0.002

## Data Availability

The original contributions presented in this study are included in the article/Supplementary Material. Further inquiries can be directed to the corresponding author(s).

## Supplementary Materials

The following supporting information can be downloaded at https://www.mdpi.com/article/10.3390/vetsci12100925/s1. Table S1: Questions related to the predictors of biosecurity. Table S2: Questions related to biosecurity compliance. Table S3: Questionnaire responses analyzed. Table S4: Results of the predictor screening using a univariate logistic regression.

## Abbreviations

The following abbreviations are used in this manuscript:

AIC	Akaike Information Criterion
HPAI	HPAI Highly Pathogenic Avian Influenza
IPPE	International Production and Processing Expo
NPIP	National Poultry Improvement Plan
U.S.	United States of America
USA	United States of America
USDA	United States Department of Agriculture
