# Supplementary material for: Socio-Psychological and External Factors Influencing Biosecurity Compliance in U.S. Poultry Farming
Source: Vet Sci. 2025 Sep 24;12(10):925. doi: 10.3390/vetsci12100925 (PMC12567621; doi:10.3390/vetsci12100925)
Supplement: Supplementary file 1 [file vetsci-12-00925-s001.zip › vetsci-3859723-supplementary.docx]

Supplementary material

**Table S1**: Questions related to the predictors of biosecurity

| **Predictors** | | | | | |
| --- | --- | --- | --- | --- | --- |
| **Conceptual**  **domains** | **Question number** | **Question formulation** | **Answer options** | **Variable code** | **Variable type** |
| Demographics | 1 | Age (optional) | (Open-ended response) | age | Numeric  (continuous) |
|  | 2 | Gender (optional) | - Female  - Male - Prefer not to say | gender | Categorical  (nominal) |
|  | 3 | Do you operate your business within the United States? | - Yes, we are a domestic entity - No, we are an international entity - Other (please specify) | us_operation | Categorical  (nominal) |
| Farm characteristics | 4 | What type of poultry do you own? | - Chickens - Ducks - Turkey - Geese - Quail - Other (please specify) | poultry_type | Categorical  (nominal) |
|  | 5 | In which of these main categories of poultry farming do you mainly belong? | - Large Producer (> 5,000 birds) - Small Producer (< 5,000 birds) - Fancy bird producer - N/A | farming_category | Categorical  (nominal) |
|  | 6 | What type of large poultry producer is your business? | - Egg Production - Broiler production - Primary Breeder - Pullet Farm - Hatchery | large_producer | Categorical  (nominal) |
|  | 7 | Do you work independently, or are you associated with a larger company? | - Associated with another company - Independently | independence | Categorical  (binary) |
| Farming Experience | 8 | How many years of experience in poultry farming do you have? | - Up to 5 years - 5 to 10 years - 10 to 20 years - 20+ years | experience | Categorical  (nominal) |
| Risk & Impact perception | 9 | Have you ever faced a major challenge related to avian diseases in your business? | - Yes (please specify) - No | major_chalange | Categorical  (binary) |
|  | 10 | Briefly explain which disease and how it impacted your activity | (Open-ended response) | disease_chalange | Qualitative  (text) |
|  | 11 | How would you assess the impact on your business in the case of an outbreak of an avian disease, such as Avian Influenza? | - Economic Losses: 1 (Low) to 10 (High)- | Impact_economic | Numeric  (scale) |
|  |  |  | Reputation: 1 (Low) to 10 (High) | Impact_reputation | Numeric  (scale) |
| Contextual Influences | 12 | Do you agree that farmers are jointly responsible for preventing animal diseases to protect both: | - Public Health: 1 (Low) to 10 (High) | responsibility_public | Numeric  (scale) |
|  |  |  | - Domestic avian population in your business: 1 (Low) to 10 (High) | responsibility_sector | Numeric  (scale) |
|  | 13 | Does any of your business partners require higher biosecurity standards than those recommended by national authorities? | - Yes - No | partner_standards | Categorical  (binary) |
|  | 14 | Do you receive the support you need for the implementation of these standards? | - Yes - No | partner_standards_support | Categorical  (binary) |
| Knowledge | 15 | How much do you agree with the statement "I receive all the necessary information and advice about the measures that I need to take to prevent the introduction of avian diseases, such as Avian Influenza, into my farm, from the..." Public sector: | - National Animal Health Authorities: 1 (Low) to 10 (High) | necessary_info_public_Authorities | Numeric  (scale) |
|  |  |  | County Extension Agents: 1 (Low) to 10 (High) | necessary_info_public_extension_agents | Numeric  (scale) |
|  |  | How much do you agree with the statement "I receive all the necessary information and advice about the measures that I need to take to prevent the introduction of avian diseases, such as Avian Influenza, into my farm, from the..." Private sector: | Cooperatives/Federations/Associations: 1 (Low) to 10 (High) | necessary_info_private_associations | Numeric  (scale) |
|  |  |  | - Integrators: 1 (Low) to 10 (High) | necessary_info_private_integrators | Numeric  (scale) |
|  | 16 | Whose opinion do you value the most when it comes to disease prevention and control? | - My own, based on experience - My own, based on research (please briefly list sources) - External inputs (please briefly list) - Consultation with other people (please briefly list) | opinion | Categorical  (nominal),  Qualitative  (text) |

**Table S2**: Questions related to biosecurity compliance

| **Biosecurity** | | | | |
| --- | --- | --- | --- | --- |
| **Topic** | **Question number** | **Question**  **formulation** | **Answer options** | **Variable code** |
| Biosecurity audit | 17 | Has a National Poultry Improvement Plan (NPIP) audit ever been conducted in your business? | - Yes - No - N/A | npip |
| Biosecurity planning | 18 | Do you follow a biosecurity plan specifically designed for your business? | - Yes - No - N/A | biosecurity_plan |
|  | 19 | Is the biosecurity plan you follow reviewed every year? | - Yes - No - N/A | biosecurity_plan_review |
|  | 20 | When was the last time? | (Open-ended response) | biosecurity_plan__last_review |
| Training | 21 | Does your staff have easy access to biosecurity training materials? | - Yes - No - N/A | staff_training |
|  | 22 | If yes, describe which? | (Open-ended response) | staff_training_open |
| Farm infrastructure | 23 | Does your poultry have access to the outdoors? | - Yes - No - N/A | outdoor_access |
| Clothes and disinfection | 24 | When your staff enters the poultry house(s), do they follow clear hygienic procedures? | - (1) No, and there is no intention to do it - (2) No, but it's being considered - (3) Yes, but only sometimes - (4) Yes, always - N/A | staff_enter_hygienic |
|  | 25 | When non-farm personnel enter the premises, do they follow a clear set of protective measures? | - (1) No, and there is no intention to do it - (2) No, but it's being considered - (3) Yes, but only sometimes - (4) Yes, always - N/A | non_farm_personnel_hygienic |
| Personnel contacts and other birds | 26 | If a staff member had contact with other poultry species, do they follow special procedures before re-entering? | - (1) No, and there is no intention to do it - (2) No, but it's being considered - (3) Yes, but only sometimes - (4) Yes, always- N/A | staff_poultry_contact |
| Contact with wild birds | 27 | Are there control measures to prevent poultry from contacting wild birds, feces, or feathers? | - (1) No, and no plans to do so - (2) No, but plans to do so - (3) Yes, but partially applied - (4) Yes, measures are in place and always applied  - N/A | wild_birds |
| Contact with Rodents and Insects, other animals | 28 | Are there control measures to protect poultry from rodents, insects, and other animals? | - (1) No, and no plans to do so  - (2) No, but plans to do so  - (3) Yes, but partially applied  - (4) Yes, measures are in place and always applied  - N/A | rodents_insects_others |
| Equipment | 29 | Are there control measures to ensure cleaning and disinfection of farm equipment? | - (1) No, and no plans to do so  - (2) No, but plans to do so  - (3) Yes, but partially applied  - (4) Yes, measures are in place and always applied  - N/A | farm_equipment_ disinfection |
|  | 30 | Do you allow the use of shared farm equipment? | From other farms:  -Yes  -No  - N/A | shared_equipment_other_farms_ |
|  |  |  | Between poultry houses:  - Yes - No - N/A | cshared_equipment_between_houses |
| Replacement Poultry– Trusted sources | 31 | If you purchase replacement poultry, do you select sources that comply with NPIP provisions? | - (1) No, and no intention to do it - (2) No, but it's being considered - (3) Yes, but only sometimes - (4) Yes, always- N/A | replacement_poultry |
| Water supply | 32 | Do your birds have direct access to surface water? | - Yes - No - N/A | surface_water |
|  | 33 | If you use water from a surface source, is the water treated? | - Yes - No - N/A | surface_water_treatment |
| Feed | 34 | Do you store feed and feed ingredients in closed containers? | - (1) No, and no intention to do it  - (2) No, but it's being considered  - (3) Yes, but only sometimes  - (4) Yes, always  - N/A | feed_containers |
| Beeding materials | 35 | Do you store replacement bedding materials? | - Yes (answer Q36)  - No (move to Q37)  - N/A | bedding_materials_store |
|  | 36 | Do you store replacement bedding materials in closed containers? | - (1) No, and no intention to do it  - (2) No, but instructions to do it  - (3) Yes, but only sometimes  - (4) Yes, always  - N/A | bedding_materials_containers |
| Sick Birds | 37 | Does your staff know which procedures to follow in case they find a sick bird? | - (1) No, none of them do  - (2) No, most of them do not  - (3) Yes, most of them do  - (4) Yes, all of them do  - I don't know  - N/A | sick_bird_report |

**Table S3:** Questionnaire responses analyzed

| **Predictors** | | | | | |
| --- | --- | --- | --- | --- | --- |
| **Question number** | **Variable** | **Mean (SD)** |  | **Valid answers  (%of total)** | **Missing answers  (%of total)** |
| 1 | age | 36.9 (14.7) |  | 38 (56.7%) | 29 (43.3%) |
| **Question number** | **Variable** | **Answer** | **Frequency  (%of valid answers)** | **Valid answers  (%of total)** | **Missing answers  (%of total)** |
| 2 | gender | Female | 20 (47.6%) | 42 (62.7%) | 25 (37.3%) |
|  |  | Male | 21 (50.0%) |  |  |
|  |  | Prefer not to say | 1 (2.4%) |  |  |
| 3 | us_operation | Yes, we are a domestic entity | 53 (81.5%) | 65 (97.0%) | 2 (3.0%) |
|  |  | No, we are an international entity | 9 (13.8%) |  |  |
|  |  | Not a buisness | 3 (4.6%) |  |  |
| 3 | us_operation_other | Backyard flock owner | 1 (33.3%) | 3 (4.5%) | 64 (95.5%) |
|  |  | Hobby farm | 1 (33.3%) |  |  |
|  |  | Small flock owner | 1 (33.3%) |  |  |
| 4 | poultry_type | Chickens | 59 (88.1%) | 67 (100.0%) | 0 (0.0%) |
|  |  | Ducks | 1 (1.5%) |  |  |
|  |  | Multispecies (previously other) | 3 (4.5%) |  |  |
|  |  | Turkey | 4 (6.0%) |  |  |
| 4 | poultry_type_other | Chickens and Turkeys | 1 (33.3%) | 3 (4.5%) | 64 (95.5%) |
|  |  | Chickens, Ducks, Turkeys | 2 (66.6%) |  |  |
| 5 | farming_category | Large producer (more than 5,000 birds) | 38 (61.2%) | 62 (92.5%) | 5 (7.5%) |
|  |  | Small producer (less than 5,000 birds) | 16 (25.8%) |  |  |
|  |  | Fancy bird producer | 8 (12.9%) |  |  |

| 6 | large_producer | Broiler Production | 19 (50.0%) | 38 (56.7%) | 29 (43.3%) |
| --- | --- | --- | --- | --- | --- |
|  |  | Egg Production | 13 (34.2%) |  |  |
|  |  | Hatchery | 5 (13.2%) |  |  |
|  |  | Primary Breeder | 1 (2.6%) |  |  |
| 7 | independence | Associated with another company | 19 (33.3%) | 57 (85.0%) | 10 (15.0%) |
|  |  | Independent | 38 (66.7%) |  |  |
| 8 | experience | < 5 years | 25 (37.9%) | 66 (98.5%) | 1 (1.5%) |
|  |  | 5 - 10 years | 13 (19.7%) |  |  |
|  |  | 10 - 20 years | 9 (13.6%) |  |  |
|  |  | > 20 years | 19 (28.8%) |  |  |
| 9 | major_chalange | Yes | 23 (36.5%) | 63 (94.0%) | 4 (6.0%) |
|  |  | No | 40 (63.5%) |  |  |
| 10 | disease_chalange | Avian influenza-related | 6 (28.6%) | 21 (31.3%) | 46 (68.7%) |
|  |  | Other avian diseases | 15 (71.4%) |  |  |
| **Question number** | **Variable** | **Min ≤ med ≤ max** | **Mean (SD)** | **Valid answers  (%of total)** | **Missing answers  (%of total)** |
| 11 | Impact_economic | 0 ≤ 8 ≤ 10 | 6.6 (3.1) | 63 (94.0%) | 4 (6.0%) |
| 11 | Impact_reputation | 0 ≤ 5 ≤ 10 | 4.9 (3.0) | 58 (86.6%) | 9 (13.4%) |
| 12 | responsibility_public | 0 ≤ 8 ≤ 10 | 7.8 (2.4) | 65 (97.0%) | 2 (3.0%) |
| 12 | responsibility_sector | 0 ≤ 8 ≤ 10 | 7.3 (2.6) | 58 (86.6%) | 9 (13.4%) |
| **Question number** | **Variable** | **Answer** | **Frequency**  **(%of valid answers)** | **Valid answers**  **(%of total)** | **Missing answers**  **(%of total)** |
| 13 | partner_standards | No | 28 (50.9%) | 55 (82.1%) | 12 (17.9%) |
|  |  | Yes | 27 (49.1%) |  |  |
| 14 | partner_standards_support | No | 4 (15.4%) | 26 (38.8%) | 41 (61.2%) |
|  |  | Yes | 22 (84.6%) |  |  |

| **Question number** | **Variable** | **Min ≤ med ≤ max** | **Mean (SD)** | **Valid answers**  **(%of total)** | **Missing answers**  **(%of total)** |
| --- | --- | --- | --- | --- | --- |
| 15 | necessary_info_public_Authorities | 0 ≤ 8 ≤ 10 | 7.5 (2.5) | 65 (97.0%) | 2 (3.0%) |
| 15 | necessary_info_public_extension_agents | 0 ≤ 8 ≤ 10 | 7.0 (2.6) | 65 (97.0%) | 2 (3.0%) |
| 15 | necessary_info_private_associations | 0 ≤ 8 ≤ 10 | 7.1 (3.1) | 64 (95.5%) | 3 (4.5%) |
| 15 | necessary_info_private_integrators | 0 ≤ 8 ≤ 10 | 7.0 (3.1) | 63 (94.0%) | 4 (6.0%) |
| **Question number** | **Variable** | **Answer** | **Frequency**  **(%of valid answers)** | **Valid answers  (%of total)** | **Missing answers  (%of total)** |
| 16 | opinion | Consultation with other people | 17 (25.8%) | 66 (98.5%) | 1 (1.5%) |
|  |  | External inputs | 10 (15.2%) |  |  |
|  |  | My own, based on experience | 31 (47.0%) |  |  |
|  |  | My own, based on research | 8 (12.1%) |  |  |
| **Question number** | **Variable** | **Answer (grouped)** | **Frequency  (%of participants that mentioned)** | **Valid answers  (%of total)** | **Missing answers  (%of total)** |
| 16 | opinion _sources | Government and Regulatory Authorities | 9 (33%) | 27 (40.2%) | 40 (59.8%) |
|  |  | Industry and Commercial Sources | 8 (30%) |  |  |
|  |  | Veterinary and Animal Health professionals | 7 (26%) |  |  |
|  |  | Research and Academic Institutions | 6 (22%) |  |  |
|  |  | Online news | 3 (11%) |  |  |
| **Biosecurity** | | | | | |
| **Question number** | **Variable** | **Answer** | **Frequency  (%of valid answers)** | **Valid answers**  **(%of total)** | **Missing answers  (%of total)** |
| 17 | npip | Yes | 33 (56.9%) | 58 (86.6%) | 9 (13.4%) |
|  |  | No | 25 (43.1%) |  |  |
| 18 | biosecurity_plan | Yes | 57 (89.0%) | 64 (97.0%) | 3 (3.0%) |
|  |  | No | 7 (11.0%) |  |  |

| 19 | biosecurity_plan_review | Yes | 53 (88.3%) | 60 (89.6%) | 7 (10.4%) |
| --- | --- | --- | --- | --- | --- |
|  |  | No | 7 (11.7%) |  |  |
| 20 | biosecurity_plan__last_review | 2 years | 2 (40.0%) | 5 (7.5%) | 60 (92.5%) |
|  |  | 3 years | 1 (20.0%) |  |  |
|  |  | 4 years | 1 (20.0%) |  |  |
| 21 | staff_training | Yes | 41 (85.4%) | 48 (71.6%) | 19 (28.4%) |
|  |  | No | 7 (14.6%) |  |  |
| **Question number** | **Variable** | **Answer (grouped)** | **Frequency  (%of participants that mentioned)** | **Valid answers**  **(%of total)** | **Missing answers  (%of total)** |
| 22 | staff_training_open | Online and Digital Resources | 6 (33.3%) | 18 (26.9%) | 49 (73.1%) |
|  |  | Internal Resources | 8 (44.4%) |  |  |
|  |  | Public and Government Resources | 4 (22.2%) |  |  |
| **Question number** | **Variable** | **Answer** | **Frequency  (%of valid answers)** | **Valid answers**  **(%of total)** | **Missing answers  (%of total)** |
| 23 | outdoor_access | Yes | 30 (45.5%) | 66 (98.5%) | 1 (1.5%) |
|  |  | No | 36 (54.5%) |  |  |
| 24 | staff_enter_hygienic | No, and there is no intention to do so | 3 (5.1%) | 59 (88.1%) | 8 (11.9%) |
|  |  | No, but it's being considered | 4 (6.8%) |  |  |
|  |  | Yes, but only sometimes | 5 (8.5%) |  |  |
|  |  | Yes, always | 47 (79.7%) |  |  |
| 25 | non_farm_personnel_hygienic | No, and there is no intention to do so | 4 (7.1%) | 56 (83.6%) | 11 (16.4%) |
|  |  | No, but it's being considered | 4 (7.1%) |  |  |
|  |  | Yes, but only sometimes | 6 (10.7%) |  |  |
|  |  | Yes, always | 42 (75.0%) |  |  |

| 26 | staff_poultry_contact | No, and there is no intention to do so | 4 (6.9%) | 58 (86.6%) | 9 (13.4%) |
| --- | --- | --- | --- | --- | --- |
|  |  | No, but it's being considered | 5 (8.6%) |  |  |
|  |  | Yes, but only sometimes | 4 (6.9%) |  |  |
|  |  | Yes, always | 45 (77.6%) |  |  |
| 27 | wild_birds | No, and there are no plans to do so | 5 (8.3%) | 60 (89.6%) | 7 (10.4%) |
|  |  | No, but there are plans to do so | 8 (13.3%) |  |  |
|  |  | Yes, but these are on only partially applied | 6 (10.0%) |  |  |
|  |  | Yes, measures are in in place and are always applied | 41 (68.3%) |  |  |
| 28 | rodents_insects_others | No, and there are no plans to do so | 2 (3.2%) | 62 (92.5%) | 5 (7.5%) |
|  |  | No, but there are plans to do so | 3 (4.8%) |  |  |
|  |  | Yes, but these are on only partially applied | 10 (16.1%) |  |  |
|  |  | Yes, measures are in in place and are always applied | 47 (75.8%) |  |  |
| 29 | farm_equipment_disinfection | No, and there are no plans to do so | 5 (7.9%) | 63 (94.0%) | 4 (6.0%) |
|  |  | No, but there are plans to do so | 2 (3.2%) |  |  |
|  |  | Yes, but these are on only partially applied | 8 (12.7%) |  |  |
|  |  | Yes, measures are in in place and are always applied | 48 (76.2%) |  |  |
| 30 | shared_equipment_other_farms | Yes | 11 (17.2%) | 64 (95.5%) | 3 (4.5%) |
|  |  | No | 53 (82.8%) |  |  |
| 30 | shared_equipment_between_houses | Yes | 25 (42.4%) | 59 (88.1%) | 8 (11.9%) |
|  |  | No | 34 (57.6%) |  |  |
| 31 | replacement_poultry | No, and there is no intention to do so | 7 (14.0%) | 50 (74.6%) | 17 (25.4%) |
|  |  | No, but it's being considered | 6 (12.0%) |  |  |
|  |  | Yes, but only sometimes | 6 (12.0%) |  |  |
|  |  | Yes, always | 31 (62.0%) |  |  |
| 32 | surface_water | Yes | 21 (33.9%) | 62 (92.5%) | 5 (7.5%) |
|  |  | No | 41 (66.1%) |  |  |
| 33 | surface_water_treatment | Yes | 25 (71.4%) | 35 (52.2%) | 32 (47.8%) |
|  |  | No | 10 (28.6%) |  |  |
| 34 | feed_containers | No, and there is no intention to do so | 4 (6.5%) | 62 (92.5%) | 5 (7.5%) |
|  |  | No, but it's being considered | 4 (6.5%) |  |  |
|  |  | Yes, but only sometimes | 4 (6.5%) |  |  |
|  |  | Yes, always | 50 (80.6%) |  |  |
| 35 | bedding_materials_store | Yes | 41 (71.9%) | 57 (85.1%) | 10 (14.9%) |
|  |  | No | 16 (28.1%) |  |  |
| 36 | bedding_materials_containers | No, and there are no intentions to do so | 8 (19.5%) | 41 (61.2%) | 26 (38.8%) |
|  |  | No, but there are instructions to do it | 3 (7.3%) |  |  |
|  |  | Yes, but only sometimes | 5 (12.2%) |  |  |
|  |  | Yes, always | 25 (61.0%) |  |  |
| 37 | sick_bird_report | No, none of them do | 3 (5.4%) | 56 (83.6%) | 11 (16.4%) |
|  |  | Yes, most of them do | 11 (19.6%) |  |  |
|  |  | Yes, all of them do | 39 (69.6%) |  |  |
|  |  | I don't know | 3 (5.4%) |  |  |

**Table S4:** Results of the predictor screening using a univariate logistic regression

| **Model** | **Pr(>Chi)^1^** |
| --- | --- |
| gender | 0.736 |
| us_operation | **0.192** |
| poultry_type | 0.898 |
| producer_categories | 0.319 |
| **independence** | **0.188** |
| **experience** | **0.100** |
| major_chalange | 0.809 |
| **impact_comp** | **0.075** |
| responsibility_comp | 0.448 |
| partner_standards_and_support | 0.327 |
| necessary_info_public_comp | 0.945 |
| necessary_info_private_comp | 0.355 |
| **opinion** | **0.048** |

*^1^Pr(>Chi)* is the p-value testing the association between each predictor and the outcome using univariate logistic regression.
